# Supplementary material for: Fatigue during the COVID-19 pandemic: Evidence of social distancing adherence from a panel study of young adults in Switzerland
Source: PLoS One. 2021 Dec 10;16(12):e0261276. doi: 10.1371/journal.pone.0261276 (PMC8664223; doi:10.1371/journal.pone.0261276)
Supplement: S1 Table — (DOCX) [file pone.0261276.s001.docx]

**S1 Table. List of variables and question wording**

| Variable | Question | Answers categories |
| --- | --- | --- |
| Staying at home | To what extent have you adhered to the appeal to stay at home as much as possible during the last four weeks? | - I did not adhere to it at all - I have rather rarely adhered to it - I have partially adhered to it - I have mostly adhered to it - I strictly adhered to it |
| Making exceptions | Did you occasionally make exceptions and left the house even if it was not necessary? | - Never - Rarely - Once in a while - Often - Very often |
| Number of people met | And how many people from your close or extended network of friends and family members have you met in your leisure time during the last week? | *Open numeric* |
| Individual risk | How dangerous do you think would a Corona infection be for yourself?  The value 0 means "not at all dangerous" and the value 10 means "extremely dangerous". | 11-poit scale   - Lowest: Not at all dangerous - Highest: Extremely dangerous |
| Social risk | How dangerous do you think the Corona epidemic is for the health of the Swiss population?  The value 0 means "not at all dangerous" and the value 10 means "extremely dangerous". | 11-poit scale   - Lowest: Not at all dangerous - Highest: Extremely dangerous |
| Household risk | In the past 4 weeks, did you live with persons in the household who belong to the risk group? | - Yes - No |
| Pro-Social: Donation | As a thank you for participation in our study, you will receive 10 Swiss francs. You can donate part of the 10 CHF. If you donate, the rest (10 minus the donation) will be transferred to your University credit card. Would you like to donate? | - Yes, I will donate part of my 10 francs. - No, I do not donate. |
| Descriptive norm:  Staying at home of  acquaintances | And what do you think: To what extent have your acquaintances and friends adhered to the recommendation to stay at home as much as possible during the last four weeks? | - They have never adhered to it - They have mostly not adhered to it - They have partially adhered to it and partially not - They have mostly adhered to it - They have always adhered to it |
| Trust in politics | One can have varying degrees of trust in the institutions of a society. On a scale from 0 (no trust at all) to 10 (very much trust), how much trust do you place into the following institutions?   - Politics | 11-poit scale   - Lowest: No trust at all - Highest: Very much trust |
| Different coronavirus measures | To what extent do you support the following measures to curb the Corona epidemic in the second lockdown?   - Maintain distance of two meters from other people - Wear protective mask - Regular hand washing - Closure of schools - Closure of universities - Closure of restaurants and bars - Closure of non-food stores - Closure of recreational facilities (e.g. sports club, cinema, museum) - Prohibition of meetings with more than 5 people - Reduction of the public transport offer - Entry restrictions | - I do not support it at all - I rather do not support it - I partially agree with it - I rather support it - I support it very much |
