# Supplementary material for: Fatigue during the COVID-19 pandemic: Evidence of social distancing adherence from a panel study of young adults in Switzerland
Source: PLoS One. 2021 Dec 10;16(12):e0261276. doi: 10.1371/journal.pone.0261276 (PMC8664223; doi:10.1371/journal.pone.0261276)
Supplement: S2 Table — (DOCX) [file pone.0261276.s002.docx]

**S2 Table. Descriptive Information of variables of wave two (2021)**

| Variable | Obs | Min | Max | Mean | SD | Answer categories |
| --- | --- | --- | --- | --- | --- | --- |
| Staying at home | 364 | 1 | 5 | 3.5 | 0.997 | 1 = not at all *to*  5 = very strictly |
| Making exceptions | 364 | 1 | 5 | 3.0 | 0.97 | 1 = very often *to*  5 = never |
| Number of people met | 364 | 0 | 47 | 6.78 | 5.31 | Open numeric |
| Individual risk | 364 | 0 | 10 | 3.24 | 2.22 | 0 = not at all dangerous *to* 10 = extremely dangerous |
| Social risk | 364 | 0 | 10 | 5.67 | 1.94 | 0 = not at all dangerous *to* 10 = extremely dangerous |
| Household risk | 364 | 0 | 1 | 0.28 |  | 0 = no high-risk person in household, 1 = living with high-risk person |
| Sex | 364 | 0 | 1 | 0.64 |  | 0 = male, 1 = female |
| Age | 364 | 19 | 36 | 24.22 | 3.11 | Open numeric birthday |
| Pro-Social: Donation | 364 | 0 | 1 | 0.52 |  | 0 = no donation,  1 = donation |
| Descriptive norm:  Staying at home of  acquaintances | 364 | 1 | 5 | 3.12 | 0.77 | 1 = not at all *to*  5 = very strictly |
| Trust in politics | 364 | 0 | 9 | 5.25 | 1.99 | 0 = no trust at all *to*  10 = very much trust |
| Social desirability | 364 | 0 | 10 | 6.18 | 1.91 | 0 = no social desirability *to* 10 = high social desirability |
| M1 Maintenance of social distance | 364 | 1 | 5 | 4.38 | 0.8 | 1 = agree not at all *to*  5 = agree very much |
| M2 Wearing of masks | 364 | 1 | 5 | 4.42 | 0.92 | 1 = agree not at all *to*  5 = agree very much |
| M3 Thorough handwashing | 364 | 2 | 5 | 4.81 | 0.5 | 1 = agree not at all *to*  5 = agree very much |
| M4 Closure of schools | 364 | 1 | 5 | 2.47 | 1.16 | 1 = agree not at all *to*  5 = agree very much |
| M5 Closure of universities | 364 | 1 | 5 | 3.4 | 1.23 | 1 = agree not at all *to*  5 = agree very much |
| M6 Closure of restaurants/ bars | 364 | 1 | 5 | 3.13 | 1.27 | 1 = agree not at all *to*  5 = agree very much |
| M7 Closure of non-food shops | 364 | 1 | 5 | 3.04 | 1.23 | 1 = agree not at all *to*  5 = agree very much |
| M8 Closure of recreational facilities | 364 | 1 | 5 | 2.96 | 1.23 | 1 = agree not at all *to*  5 = agree very much |
| M10 Meetings of only 5 or  fewer | 364 | 1 | 5 | 4.15 | 1.25 | 1 = agree not at all *to*  5 = agree very much |
| M11 Restrictions to public transport | 364 | 1 | 5 | 2.58 | 1.26 | 1 = agree not at all *to*  5 = agree very much |
| M12 Border restrictions | 364 | 1 | 5 | 3.59 | 1.3 | 1 = agree not at all *to*  5 = agree very much |

Obs = number of observations, min = minimum, max = maximum, SD = standard deviation
