# Supplementary material for: Fatigue during the COVID-19 pandemic: Evidence of social distancing adherence from a panel study of young adults in Switzerland
Source: PLoS One. 2021 Dec 10;16(12):e0261276. doi: 10.1371/journal.pone.0261276 (PMC8664223; doi:10.1371/journal.pone.0261276)
Supplement: S3 Table — (DOCX) [file pone.0261276.s003.docx]

**S3 Table. List of index variables for the first wave (2020), and the second wave (2021).**

| Variable | Obs | Min | Max | Mean | SD | Description |
| --- | --- | --- | --- | --- | --- | --- |
| Index Compliance with social distancing measures 2021 | 364 | 0.61 | 11.87 | 6.54 | 2.13 | Additive index containing 2 distancing measures (each on a 5-point Likert scale, ranging from 0 to 4), and the reversed logarithm of the number of people met. Higher values indicate more compliance. |
| Index Compliance with social distancing measures 2020 | 364 | 1.21 | 11.04 | 7.01 | 1.57 | Additive index containing 2 distancing measures (each on a 5-point Likert scale, ranging from 0 to 4), and the reversed logarithm of the number of people met. Higher values indicate more compliance. |
| Index  Support for  Covid-19  measures 2021 | 364 | 8 | 40 | 27.03 | 6.43 | Additive index containing 8 preventive measures (each on a 5-point Likert scale, ranging from 1 to 5). Higher values indicate stronger support. |
| Index  Support for  Covid-19  measures 2020 | 364 | 8 | 40 | 32.01 | 5.14 | Additive index containing 8 preventive measures (each on a 5-point Likert scale, ranging from 1 to 5). Higher values indicate stronger support. |

Obs = number of observations, min = minimum, max = maximum, SD = standard deviation
