# Supplementary material for: Fatigue during the COVID-19 pandemic: Evidence of social distancing adherence from a panel study of young adults in Switzerland
Source: PLoS One. 2021 Dec 10;16(12):e0261276. doi: 10.1371/journal.pone.0261276 (PMC8664223; doi:10.1371/journal.pone.0261276)
Supplement: Attachment — Submitted filename: response_to_reviewers.docx [file pone.0261276.s004.docx]

Dear Asim Zia

First of all, we thank you and the two reviewers very much for the effort of reviewing our manuscript and for the helpful suggestions. We incorporated almost all of them and respond to every comment point by point as they were made by the reviewers.

Response to reviewer 1:

1) The possibility that people met more often in 2021 as compared to 2020 because masks were viewed as preventing infections is a good comment. However, risk perception did not change between 2020 and 2021 which speaks against this possibility. Furthermore, we recalculated the panel regression model, once with the index excluding wearing face masks, and second including additionally the face masks as a separate indicator. First, results of index without face masks does not change the results. Second, treating the face masks as an additional indictor has no statistically significant effect. The table with the regression is shown below. Hence, face masks have no influence on the compliance with social distancing and there is no need for any change of the manuscript.

Table 1: Two-way fixed effects panel regression

|  | Variables | | Coefficient | | Index without masks | | Index without  + mask | |
| --- | --- | --- | --- | --- | --- | --- | --- | --- |
| Support of COVID-19 measures | | 0.34***  (0.059) | | 0.36***  (0.062) | | 0.35***  (0.063) | |  |
| Wearing of face masks  Household risk | |  | |  | | 0.04  (0.058) | |  |
| Transition to household with person at risk^§^ | | 0.35  (0.183) | | 0.35  (0.183) | | 0.35  (0.183) | |  |
| Leaving of household with person at risk^§^ | | 0.07  (0.186) | | 0.08  (0.186) | | 0.07  (0.186) | |  |
| Second Wave^§^  Constant | | -0.01  (0.073)  -0.01  (0.046) | | -0.1  (0.085)  -0.07  (0.051) | | 0.04  (0.120)  -0.04  (0.067) | |  |
| Within R^2^ | | 0.143 | | 0.143 | | 0.114 | |  |
| n | | 364 | | 364 | | 364 | |  |
| n x T | | 728 | | 728 | | 728 | |  |

Note: * = p < 0.05, ** = p < 0.01, *** = p < 0.001. Standardized regression coefficients with standard errors in brackets. § = Dichotomous variables, which are not standardized.

2) Thank you for the comment. We include the abbreviations M1 through M12 to the description of the measures on page 9 and in Figure 2. Furthermore, we include a sentence that we measure social desirability by the Marlowe-Crowne scale on page 10 and provide two new references.
3) Good comment, but please note that our study is one of the first that reports panel results comparing different lock down periods. However, we did find one study that fits into the discussion part and we refer to it in the revised version.
4) Yes, in both waves the questions referring to how much respondents stayed at home refers to a four-week period.
5) Thank you for the comment. We included the results of the significance tests in the revised version.
6) Thank you for the comment. We rephrased the title of Table 1 to “Two-way fixed effects panel regression on the change of complying with social distancing measures”.
7) Thank you for reading also the supplement. We corrected the typo.
8) We checked the numbers again. It is exactly 60.98 and we rounded to 61.0% in Fig 3. We also corrected it to 61% in the text.
9) Thank you very much for pointing out the typos which we corrected.

Response to reviewer 2:

1) Yes, thank you for the comment, we included the paper by Wändi Bruine de Bruin and Daniel Bennett (2020).
2) We added in the abstract that we have a sample of young adults from the University of Bern, Switzerland.
3) Thank you for the comment: As suggested, we removed the mentioned part from the Methods section into the Results section.
4) Thank you for the comment. We think that clarity improved by following suggestion 3. The rest of the method section describes the survey instruments (the measurement of variables), and how the variables were coded step by step. We think this is easier to comprehend instead of first describing all the questions and then the coding afterwards. We believe this way we avoid unnecessary redundancies. Similar arguments apply to the description of the statistical procedures. Since we have more than one statistical model (structural equation model and panel regression), we believe describing first each model followed by its results is easier to comprehend as compared to describing first all models and afterwards all results.
5) Thank you for this suggestion. We include the exact question wording in a new table (S1 Table) in the supporting information and refer to it in the text.
6) We followed this suggestion and moved the overview of the analyses into the method section.
7) Thank you for this suggestion. We incorporated the error bars into Fig 2.
8) This is a very helpful comment. We recalculated the model dropping three of the eleven indicators of the acceptance of the COVID-19 measures. These indicators were described as weak indicators already in the first version of the manuscript. Dropping the three indicators improved the model fit CFI and TLI to 0.957 and 0.945 respectively. None of the structural estimates was affected by this slight change of the measurement of the latent construct “support of COVID-19 measures”. We rewrote the results section accordingly, which makes it also more comprehensible.
9) We also included a few sentences explaining which relations had been tested but not found significant. The new sentences are marked in the revised version.
10) Thank for this comment. It is a good point and we considered it thoroughly. However, the model in Figure 1 is a reference of the results of the first wave. A replication of it with the data of wave 2 is presented in Figure 4. The results are very similar. Hence, it would be redundant to present the model with the data of wave 2 twice with only minor differences. Therefore, we believe it is better to leave Fig 1 as is, and we hope that the reviewer can accept our argument.
11) We did included a couple of sentence improving the explanation of the coding and construction of the index of the dependent variable of the panel regression model.
